# Advancing acceptance: assessing acceptance of the ESR iGuide clinical decision support system for improved computed tomography test justification

**DOI:** 10.3389/fmed.2023.1234597

**Published:** 2023-12-14

**Authors:** Clara Singer, Osnat Luxenburg, Shani Rosen, Sharona Vaknin, Mor Saban

**Affiliations:** ^1^Research Center for Medical Technology Policy and Innovation, The Gertner Institute for Epidemiology and Health Policy Research, The Chaim Sheba Medical Center, Tel Hashomer, Ramat Gan, Israel; ^2^Medical Technology, Health Information and Research Directorate, Ministry of Health, Jerusalem, Israel; ^3^Nursing Department, School of Health Professions, Faculty of Medicine, Tel Aviv University, Tel Aviv, Israel

**Keywords:** appropriateness criteria, appropriate imaging, clinical decision support system, radiation protection, ESR iGuide

## Abstract

**Background:**

A clinical decision support system (CDSS), the European Society of Radiologists (ESR) iGuide, was developed to address gaps in the availability and use of effective imaging referral guidelines.

**Aim:**

This study aimed to assess the appropriateness of computed tomography (CT) exams with and without ESR iGuide use, as well as the usability and acceptance of the physician systems.

**Methods:**

A retrospective single-center study was conducted in which data from 278 consecutive CT tests referred by physicians were collected in the first phase (T1), and physicians used the ESR iGuide system for imaging referrals in the second phase (T2; *n* = 85). The appropriateness of imaging referrals in each phase was assessed by two experts, and physicians completed the System Usability Scale.

**Results:**

The mean appropriateness level on a scale of 0–9 was 6.62 ± 2.69 at T1 and 7.88 ± 1.4 at T2. When using a binary variable (0–6 = non-appropriate; 7–9 = appropriate), 70.14% of cases were found appropriate at T1 and 96.47% at T2. Surgery physician specialty and post-intervention phase showed a higher likelihood of ordering an appropriate test (*p* = 0.0045 and *p* = 0.0003, respectively). However, the questionnaire results indicated low system trust and minimal clinical value, with all physicians indicating they would not recommend collegial use (100%).

**Conclusion:**

The study suggests that ESR iGuide can effectively guide the selection of appropriate imaging tests. However, physicians showed low system trust and use, indicating a need for further understanding of CDSS acceptance properties. Maximizing CDSS potential could result in crucial decision-support compliance and promotion of appropriate imaging.

## Highlights

−ESR iGuide can effectively guide the selection of the appropriate imaging tests.
−Physicians demonstrated low system trust and use.
−Understanding of systems acceptance properties can increase use and maximize their potential.

## Background

Most hospital pathways require medical imaging procedures (80%), although this raises questions regarding imaging appropriateness (1). Inappropriate use leads to medical resource waste and unnecessary radiation exposure. To overcome inappropriate imaging referral rates, the application of evidence-based imaging referral guidelines and clinical decision support systems (CDSS) have been suggested. CDSS tools aim to standardize diagnostic and referral processes based on best practices. Traditionally, such clinical decision support systems have been developed by establishing a set of analytical rules to systematically evaluate different attributes within patient input data, thereby enabling standardized assessments and recommendations. This conventional rule-based approach allows CDSS to automate guideline-adherent diagnostic and referral decisions based on structured clinical data. When integrated into electronic medical record workflows, CDSS have the potential to promote imaging appropriateness at the point of care (2, 3).

Evidence-based imaging referral guidelines have been made accessible for open-access use by the American College of Radiology (ACR) (2). However, the lack of knowledge and uptake of these guidelines remains a significant issue. Granata et al. 4 assessed the availability, use, and familiarity of referral guidelines for medical imaging in children. They concluded that effective and extensive adoption of imaging referral guidelines is lacking, with 48.6% of physicians missing knowledge about the availability and proper implementation of imaging guidelines (4). Several evidence-based modeling techniques to improve guideline uptake and to ensure appropriate imaging use have been proposed, including CDSS based on the Royal College of Radiologists guidelines, the ACR appropriateness criteria, and the European Society of Radiology (ESR) guidelines, which are derived from ACR appropriateness criteria subjected to European standards of practice modifications (2).

Implementing evidence-based CDSS in clinical practice shows promising potential for improving guideline adherence, reducing unnecessary imaging, and improving diagnostic management (5). The ESR iGuide CDSS was developed to facilitate the European Society of Radiology guidelines. However, research assessing clinical implementation success, as well as the performance of the ESR iGuide in assisting diagnostic decision-making and potentially optimizing medical resources, is currently limited.

To evaluate the potential for clinical implementation success, a recent 2022 study assessed the acceptance and reliability of the ESR iGuide by senior physicians using simulated clinical cases and compared the level of agreement with ESR iGuide’s recommended procedures. Results showed complete system recommendation agreement in 75% of cases and a 77.28% agreement between experts when considering a binary agree/disagree variable (6). In addition, a 2020 study evaluated the application of the ESR iGuide on clinical decision making within the oncology hepatocellular carcinoma and cholangiocarcinoma frameworks. The authors concluded that the ESR iGuide could help guide appropriate imaging selection and optimize medical resources by reducing inappropriate testing (5). Moreover, CDSSs such as the ESR iGuide can aid physicians with the management of both individual patients and their overall caseload. However, there is considerable evidence of low uptake and dissatisfaction with use by physicians, indicating problems with CDSS purpose and delivery (7).

To better understand what barriers hinder the use and usability of CDSSs, a qualitative study by Ford et al. was performed using thematic analysis (7). The results suggested that CDSS use was affected by provenance trust, observed threat to autonomy, and well-defined administration guidance. CDSS use was subjected to ‘user fatigue’ and effective use guidance (7). Further studies have shown that physicians lack the inclination and ability to use technological systems, potentially due to not accepting their utility, resulting in potentially reduced quality of care (8). Subsequently, theoretical technology acceptance models were developed to provide a detailed understanding of user acceptance and technology use (9). A recent task analysis (2018) using the theoretical technology acceptance framework was performed, suggesting that CDSS favorability was derived when needs and expectations from the CDSS were provided and when the system’s principal output was clear (10).

To promote physician acceptance and system use, it may be important to consider several elements when designing new CDSSs, including the importance of CDSS end-user co-development, clear system principal output, and defined practice guidance. Accordingly, our study aimed to evaluate how the ESR iGuide could impact the appropriateness of imaging referrals. Specifically, we aimed to explore if there is an improvement in the appropriateness score of imaging examinations ordered in a public medical center after physicians were exposed to the ESR iGuide recommendations. In addition, we aimed to explore the degree of acceptance of the ESR iGuide by the clinical team using the System Usability Scale (SUS).

## Methods

### Study design

A quasi-experimental study with a pre-post intervention design was conducted between May 2021 and June 2022 in a medium-sized university teaching hospital, in which approximately 6,235 CT scans are performed annually. Nationally, approximately 575,000 in-hospital CT scans are conducted.

The pre intervention phase (T1) explored the appropriateness of CT imaging referrals during regular routine practice, while in the post-intervention phase (T2), we examined the appropriateness of imaging referrals when using the ESR iGuide tool.

### The ESR iGuide system

The ESR iGuide is an online web portal that recommends the most appropriate imaging tests based on patient data, together with their level of appropriateness, estimated cost, and expected radiation exposure (2). This system was developed in 2014 and is based on the American College of Radiology (ACR) guidelines, adapted for the European guidelines.

### Sample size

Based on the literature review, we assumed an affect size of 0.4, with a confidence level of 95%. The sample size was calculated using GPower 3.1 software based on the population size and statistical requirements for models of this type. Based on a test power of 80%, a confidence interval of 95%, and a significance of *p* = 0.05, the minimal sample size was calculated to be 78 imaging referrals in each group (pre and post intervention).

### Data collection and procedure

#### Pre-intervention phase (T1)

**Figure 1:**
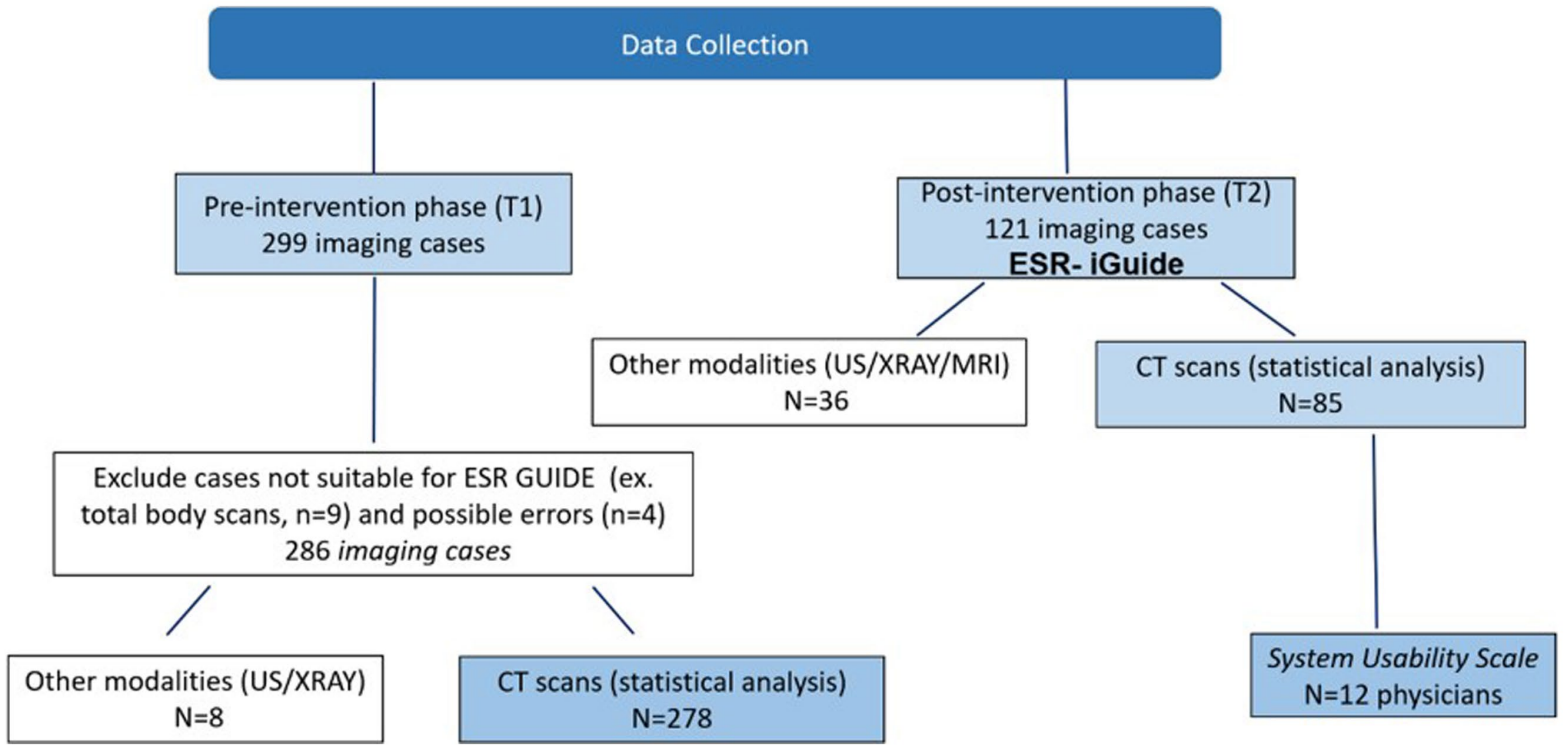
Data collection description including Pre- and Post-intervention phases.

For imaging referral cases, we collected the original text referral (clinical indications), ordered test, patient characteristics (age, gender, clinical background), and physician characteristics (gender, type of specialty, physician status – intern, resident, senior physician). We also collected data regarding the shift in which the imaging test was carried out. 278 consecutive CT imaging cases were collected in T1 (Figure 1).

Simultaneously, we examined the ESR iGuide recommendation for each scenario. For this purpose, SR and MS inserted anonymous case details into the system, including sociodemographic characteristics of the patient (age and gender), clinical indications, and red flags. The latter were defined as signs and symptoms found in the patient’s history and clinical examination that may help identify the presence of potentially serious conditions.

We then obtained the recommendations of the ESR iGuide system with the corresponding appropriateness rating grade ranging from 9 (highly recommended) to 1 (not recommended). A rating grade of 7–9 corresponded to “usually appropriate,” 4–6 was defined as “may or may not be appropriate,” and a rating of 1–3 was defined as “usually not appropriate.” We used the ESR iGuide appropriateness score of the actual exam performed. If a CT exam was not part of the recommendations, a score of zero was assigned for this analysis.

#### Post intervention phase (T2)

For this phase, we recruited physicians who agreed to use the ESR iGuide system for imaging referrals. Twelve out of thirty physicians agreed to participate. 85 consecutive CT imaging cases were performed with the assistance of the ESR iGuide system between March 2022 and June 2022 (Figure 1). For each case, the physicians inserted the relevant medical data (patient age, gender, and clinical indications) into the ESR iGuide and received the recommendations of the system. The data collection process for this phase was similarly performed to that of T1 to allow for future comparative analysis.

The appropriateness of imaging referrals in each phase was assessed by two experts. (MD and PhD with above 10 years of experience in the medical field).

#### Post-intervention phase - questionnaire

To identify usability related concerns and perspectives of the ESR iGuide, physicians were asked on a volunteer basis to complete a 13-item questionnaire based on the System Usability Scale (SUS). Items were rated on a 5-point Likert-type scale (1, “Strongly agree” to 5, “Strongly disagree”). The questionnaire was translated to Hebrew and validated by an expert group from different health organizations (*n* = 9). The alpha Cronbach was acceptable (0.81).

### Data analysis

Appropriateness scores using the ESR iGuide criteria were compared between the two study phase. In order to compare the T1 and T2 sample characteristics, descriptive statistics were computed for the following variables: original text referral (clinical indications), ordered test, patient characteristics (age, gender, clinical background), and physician characteristics (gender, type of specialty, physician status – intern, resident, senior physician) and the shift during which the scan was ordered (Morning 7:00–14:59, Evening 15:00–22:59, Night 23:00–6:59).

The correlations between the study variables, including the phase of the study (T1/T2), and the level of appropriateness were examined using Chi-square tests for categorical variables, and the appropriate *t*-tests, Pearson correlation coefficients, or one-way ANOVAs for continuous variables. A multivariate logistic regression model was used to identify variables that predict the appropriateness score. For this purpose, the ESR iGuide level of appropriateness was classified using a binary variable (scores less than 7 - non appropriate, scores between 7 and 9 - appropriate). The probability of an appropriate score was modeled.

We tested the interaction between the study phase (T1/T2) and the physician specialty (surgery / non-surgery) with ANOVA, using the appropriateness score (values 0–9) as the dependent measure.

For the System Usability Scale (SUS) questionnaire analysis, we grouped the scores into three categories: Agree: score = 1–2; Neutral: score = 3; Disagree: score = 4–5 and we calculated the percentage of responses in each of these categories.

The statistical analysis was performed using the SAS Enterprise Guide v.8.3. Significance was taken at the *p* < 0.05 level.

### Ethical considerations

The study protocol was approved by the Institutional Human Subjects Ethics Committee (CM-0058-21) of the medical facility. All the study procedures followed the ethical standards of the institutional and the national research committee, and complied with national ethical standards.

## Results

**Figure 2:**
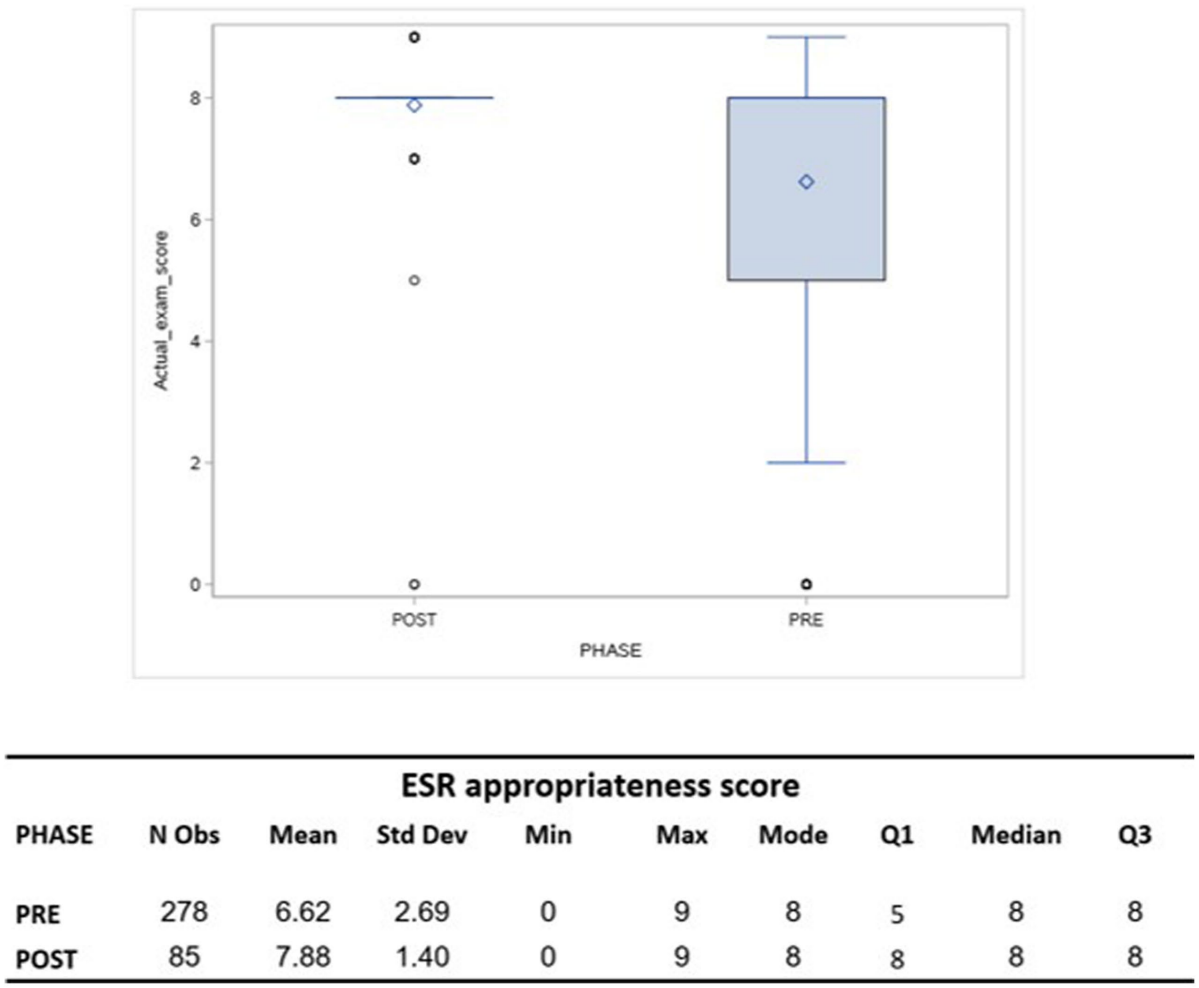
A box plot comparing the ESR appropriateness score of actual imaging test before (PRE-phase) and after (POST-phase) exposition to ESR-iGuide use.

The sample included 278 cases in T1 phase and 85 in the T2 phase. The mean age of the patients was 59.2 ± 23 years in T1 and 49.8 ± 21.5 years in T2. Non-surgery specialists were 63% in T1 and 48% in T2 (Table 1). When comparing the ESR iGuide appropriateness referral score, the overall mean of appropriateness for T1 was 6.62 ± 2.69 compared to 7.88 ± 1.4 in T2. Both samples had the same median score of 8.00 (Figure 2).

**Table 1:** Descriptive statistics of the study sample (363 CT imaging cases including pre- and post-phase).

	Pre-phase *N* = 278	Post-phase *N* = 85
**Patient characteristics**		
Age (Mean, SD)	59.2 ± 22.9	49.8 ± 21.5
**Gender**		
Female (*n*, %)	165 (59.3%)	51 (60%)
Male (*n*, %)	113 (40.6%)	32 (37.6%)
Missing		2 (2.3%)
**Physician status**		
Specialist	86 (30.9%)	12 (14.1%)
Resident/Intern	192 (69.1%)	73 (85.9%)
**Physician specialty**		
Surgery	102 (36.7%)	44 (51.8%)
Non-surgery	176 (63.3%)	41 (48.2%)
**Setting characteristics**		
*Shift (n, %)*		
Morning	80 (28.7%)	26 (30.5%)
Evening	125 (44.9%)	34 (40%)
Night	52 (18.7%)	22 (25.8%)
Missing	21 (7. 5%)	3 (3.5%)

When using a binary variable, the overall appropriate rate was 70.14% (195 out of 278 imaging referrals were considered appropriate) in T1 and 96.47% (82 out of 85) in T2 (*p* < 0.0001). The multivariate logistic regression for modeling the probability of an appropriate score suggested that T2 (post-intervention phase) and physician specialty (surgery/non-surgery) were significant (*p* = 0.0003 and *p* = 0.0045, respectively). The findings indicate that a non-surgical specialist is 0.4 times less likely to order an appropriate test according to the ESR iGuide (95% CI 0.219–0.757) compared to a surgical specialist. Furthermore, we found that physicians in T2 were 8.977 times more likely to order an appropriate test as compared to in T1 (95% CI 2.702–29.82). Age, gender, status of the physician, and shift during which the scan was ordered were not found be statistically significant (Table 2).

**Table 2:** Full Model logistic regression modelling the probability of the actual imaging test having an appropriate ESR iGuide score (score between 7 and 9).

	Analysis of maximum likelihood estimates					Odds ratio estimates and profile-likelihood confidence intervals		
	DF	Estimate	Standard error	Wald Chi-square	Pr > Chi-square	Odds ratio	95% CI OR	
							Lower	Upper
**Predictors**								
Intercept	1	1.5523	0.5523	8.833	0.033			
Age	1	0.002	0.006	0.087	0.768	1.002	0.990	1.014
Gender (male vs. female)	1	0.017	0.281	0.0038	0.951	1.018	0.586	1.766
Phase (post vs. pre)	1	2.194	0.613	12.83	0.0003*	8.977	2.702	29.82
Specialty (non-surgery vs. surgery)	1	−0.899	0.317	8.067	0.0045*	0.407	0.219	0.757
Status (specialist vs. resident/ intern)	1	−0.41	0.313	1.719	0.189	0.664	0.360	1.225
Shift (evening vs. night)	1	−0.039	0.365	0.012	0.914	0.961	0.470	1.965
Shift (morning vs. night)	1	0.019	0.401	0.002	0.962	1.019	0.464	2.237

^*^Statistically significant at the level of *p* ≤ 0.05.

**Figure 3:**
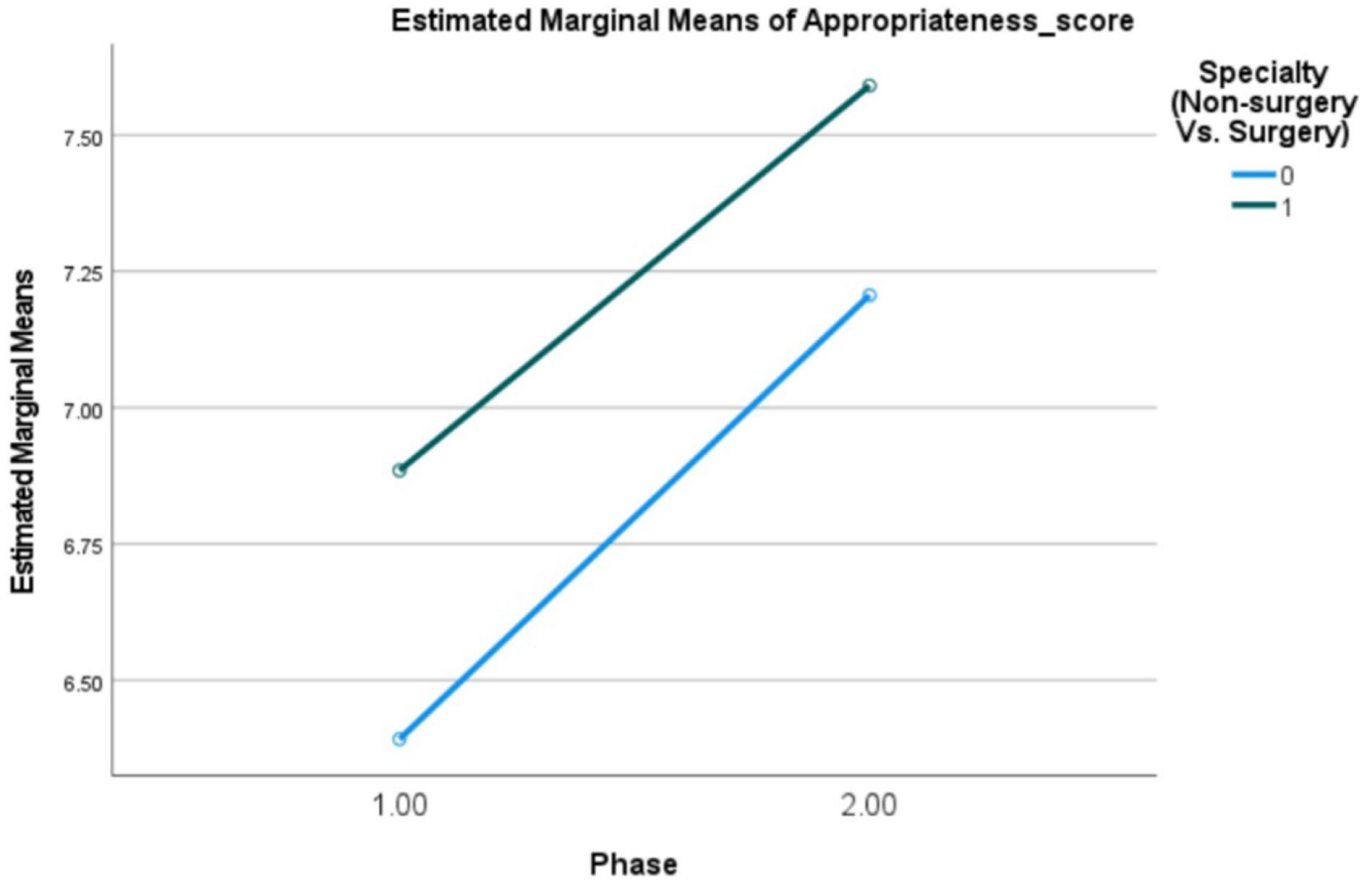
Interaction plot (PRE- 1, POST-2) Estimating and testing the interaction between the study phase (PRE- 1, POST-2) and physician specialty (Non-surgery Vs. Surgery) using ANOVA for modelling the appropriateness score (0–9).

Figure 3 and Table 3 depicts the interaction between the study phase (T1/T2) and physician specialty (surgery/non-surgery), using the appropriateness score (values 0–9) as the dependent variable. This interaction was not found to be statistically significant.

**Table 3:** Univariate analysis (dependent measure: appropriateness score) estimating and testing the interaction between the study phase (PRE- 1, POST-2) and physician specialty (non-surgery vs. surgery) using ANOVA for modelling the appropriateness score (0–9).

Model summary					ANOVA					
Model	*R*	*R* ^2^	Adjusted *R*^2^	Std. error of the estimate		Sum of squares	*df*	Mean square	*F*	Sig.
1	0.151^a^	0.023	0.017	2.579	Regression	49.652	2	24.826	3.734	0.025
					Residual	2141.105	322	6.649		
					Total	2190.757	324			
2	0.151^b^	0.023	0.014	2.583	Regression	49.800	3	16.600	2.489	0.060
					Residual	2140.957	321	6.670		
					Total	2190.757	324			

^a^Predictors: (constant), specialty, phase. ^b^Predictors: (constant), specialty, phase, interaction.

Twelve physicians participated in the survey using the SUS-based questionnaire. The results revealed that 75% of the physicians disagreed with the statement “System helped me to choose the right imaging test.” Similarly, 42% disagreed with the statement “System recommendations are evidence-based,” and 92% disagreed with the statement “I felt very confident using the system.”

When asked about system functionality, 33% of the physicians agreed that they found inconsistencies in the system, while 67% disagreed with the statement “The various functions in this system were well integrated” Regarding the frequency of system use, 92% disagreed with the statement “I would like to use this system frequently.” Additionally, 100% of the physicians disagreed with the statement “I will recommend physician colleagues to use this system.”

On the usability aspect, 92% of the physicians found the system user-friendly and easy to use. Similarly, 92% disagreed with the statement “I will need to learn a lot of things before I could use this system.”

(Figure 4).

**Figure 4:**
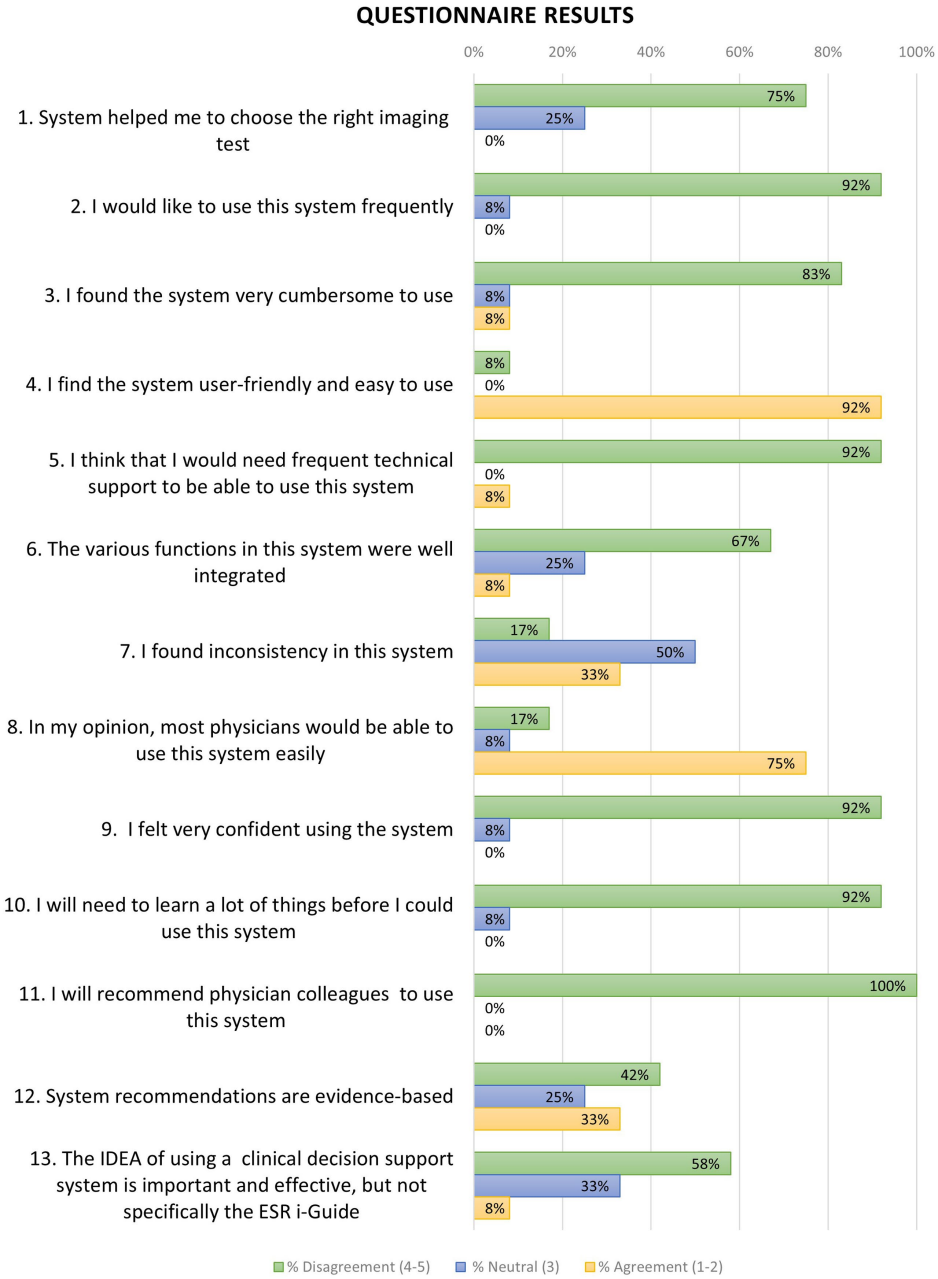
Results of 13-questions questionnaire concerning physician’s attitude towards ESR-guide use and support. Percentage of agreement (score 1–2), neutral (score 3) and disagreement (score 4–5) for each question is plotted. *N* = 12.

## Discussion

Incorporating a CDSS can assist clinicians and guide them to prescribe the most suitable test and improve patient clinical outcomes alongside optimal resource allocation (11–13). In the current study, we compared imaging referral appropriateness with and without the use of the ESR iGuide. This system is sourced on the ACR appropriateness criteria and was developed to address gaps in the availability of imaging referral guidelines and the lack of their use in Europe (14).

Although several years have passed since this initial development, their use is still debated and not completely embedded and accepted. A previous study examined the knowledge and availability of the ESR iGuide among 33,257 ESR radiology members. In total, 2067 responses (6.3%) were received from 52 countries, with only 746 (36.1%) of all respondents aware of the availability and features of the ESR iGuide (4). Israel was not part of the survey.

During 2022, the Israeli Ministry of Health (MOH) promoted an early assessment of ESR iGuide for national healthcare system use. The MOH aimed to study the readiness and acceptance among medical staff, and recommended piloting the software. This pilot study’s results suggest that the ESR iGuide can be effective in guiding the selection of appropriate imaging tests; there was an increase of 26% in referring appropriate imaging tests following the use of the ESR iGuide. Previous research by Gabelloni et al. has shown similar rates of improved appropriateness when using this system, with an expected reduction of inappropriate referrals by 20% (5).

Similarly, according to a study conducted by Salerno et al., it was found that in 45% of cases (*n* = 264 out of 587), the CT test was considered necessary using the ESR iGuide. These results highlight a concerning prevalence of unnecessary exposure to ionizing radiation (15).

Previous studies have evaluated the effectiveness of a CDSS in improving the diagnostic management of patients, showing that compared to CDSS-unassisted practice, their use can result in an increased rate of appropriate examinations and a decreased rate of inappropriate examinations (5, 16, 17). These results are in line with our study, suggesting a significant improvement in appropriate imaging referral rate.

To the best of our knowledge, there are no other published studies that have tested the practice of the ESR iGuide in assisting with imaging referral decision making and potentially optimizing healthcare resources. In addition, adoption of such CDSSs is lacking, hindering the realization of the clinical potential (18).

In the current study, we have identified features that may hinder physicians’ use of the ESR iGuide in a specified matter and as part of a broader CDSS framework. Similar features were found in previous studies and include system acceptance and a recognition of the importance of developing such CDSSs with emphasis on end-user design, user needs and expectations, principal model trust, practice context sensitivity, and certified provenance (7, 9, 10). Certified provenance, principal model trust and clinical pathway transparency are becoming more and more important in with evolving health technology; it is important that the provenance of the CDSSs is congruent with sources which are recognized as trustworthy or scientifically credible by the physician and that this provenance should be easily accessible (7, 8).

Lack of adherence to recommendations despite knowledge of such guidelines is also a significant issue. A study conducted in the primary care setting, showed that physicians followed decision-support advice for inappropriate imaging orders in only 25% of cases (20). Although the ESR iGuide is recognized as a gold standard source and the improvement in the appropriateness score was statistically significant, 92% of the physicians in our study indicated a lack in trust and reliability in the system, with 42% Disagreed that system recommendations are evidence-based. This suggests that despite the CDSSs being based on evidence-based data, their clinical use is hindered by their lack of complete development, and limited testing (8).

Moreover, despite the system being offered to the hospital at no cost, supported by two research coordinators, only a limited number of physicians agreed to utilize it, leading to a noticeable disparity in sample sizes between the two study phases. This difference can be attributed to challenges encountered during the recruitment process, likely stemming from physician reluctance and voluntary participation, which in turn suggests a potential lack of perceived value in adopting the CDSS. It underscores the critical importance of further investigating the factors that influence physician acceptance. Notably, most of the physicians that did volunteer to use the system, explicitly expressed their disinterest in integrating the system into their regular routine practice.

User acceptance theories suggest that understanding the relationship between technology and its end users determines adoption. Specifically, technology acceptance theories emphasize users’ expectations and propose that the key to adoption are the ease of use and perceived usefulness (21, 22). Indeed, uncovering the relationship between the physicians and the ESR iGuide may exhibit insight into user acceptance in practice; Among physicians in our study, 75% expressed a lack in perceived usefulness. This is contradictory to our other results suggesting ESR iGuide improves physicians’ appropriate imaging selection, indicating high system usefulness. Thus, a gap remains within the framework of user-perceived usefulness. Further enhancement of the system’s perceived usefulness in practice may be key for successful use (21, 22).

Important contributors presented in the literature for the successful use of CDSSs include technical integration into patient record systems, proper training, and system guidance (23). Thus, it is vital that CDSSs be built to complement physician knowledge, critical reasoning, and clinician autonomy (8).

In the realm of healthcare, studies suggest that the adoption of CDSSs by clinicians is influenced by meeting physicians’ expectations of technology. While a CDSS system with clinical value built on rule based principles can be effective in guiding what test may be most appropriate, there may be a growing expectation for advanced AI capabilities in today’s technologically advanced landscape (24). This may include tools capable of fostering medical critical thinking, and enhancing knowledge and professional autonomy (23).

CDSSs that increase evidence-based decision making in healthcare have the potential to improve quality of care and patient health and outcomes, but will only be of benefit if used wisely in the clinical environments (23, 25). The tension between the evidence that the CDSS improves medical decision making and physicians’ perceptions that it may not always do so, is needed. Accordingly, it is important not to dismiss this tension as an error and the emerging paradox as a fallacy. But rather, embrace it and learn from it. We hope that further development of new trustworthy and clinically transparent CDSSs together with physician empowerment to make informed decisions, critically assessing CDSS output, will contribute to enhance physician care in the age of evolving digital health.

### Study limitations

This study aimed to identify the use and usability features of the ESR-iGuide using a multi-level approach. However, there are some limitations. The case details entered were derived from medical records and may not match the actual referral inquiry. Two reviewers inserted details independently but real-world variability remains. Referral practices differ by healthcare setting, potentially introducing bias, as protocols like initial CT use for acute abdominal pain differ. The usefulness of corrections may vary across settings. We explored only one setting and recommend future studies explore practice protocol differences and CDSS effectiveness variably (15). Finally, only 12 physicians volunteered for evaluation, limiting understanding of use and usability perspectives, though 5–8 participants can identify 80% of issues (26). Our findings should be considered preliminary and help navigate effective radiology research.

## Conclusion

In conclusion, this study demonstrated the ESR iGuide’s potential to effectively guide appropriate imaging test selection. Results found recommendations aligned well with experts and helped clinicians explore options. However, further research is still needed to fully realize its clinical benefits. Larger implementations in varied healthcare settings could provide deeper insights into adoption across different user groups. Qualitative feedback from clinicians, patients, and administrators would help optimize the user experience and identify facilitators and barriers to integration. Additional predictive modeling incorporating individual patient factors may help tailor recommendations. Machine learning could further enhance accuracy over time. Standardized metrics comparing iGuide-guided to usual ordering should evaluate impacts on appropriateness, costs and quality. Addressing key implementation factors through continued refinement informed by future research may help decision support tools like the iGuide improve imaging selection and patient care.

## Data availability statement

The raw data supporting the conclusions of this article will be made available by the authors, without undue reservation.

## Ethics statement

The studies involving human participants were reviewed and approved by the Institutional Human Subjects Ethics Committee of the Gertner Institute for Epidemiology and Health Policy Research Chaim Sheba Medical Center School of Public Health, Sackler Faculty of Medicine, Tel Aviv University (CM-0058-21). Written informed consent from the participants was not required to participate in this study in accordance with the national legislation and the institutional requirements.

## Author contributions

SR, CS, and MS interpreted the data and edited and approved the final article and critically reviewed the manuscript for important intellectual content. OL, MS, and SV conceptualized and designed the study, drafted the initial manuscript, and reviewed and revised the manuscript. CS, MS, SV, and OL designed the methods section, analyzed the data and reviewed and revised the manuscript. All authors contributed to the article and approved the submitted version.
